# Noninvasive analysis of contractility during identical maturations revealed two phenotypes in ventricular but not in atrial iPSC-CM

**DOI:** 10.1152/ajpheart.00527.2023

**Published:** 2024-01-05

**Authors:** Marcel Rapöhn, Lukas Cyganek, Niels Voigt, Gerd Hasenfuß, Stephan E. Lehnart, Jörg W. Wegener

**Affiliations:** ^1^Department of Cardiology and Pulmonology, University Medical Center of Göttingen, Göttingen, Germany; ^2^German Centre for Cardiovascular Research (Deutsches Zentrum für Herz-Kreislaufforschung), Göttingen, Germany; ^3^Department of Pharmacology and Toxicology, University Medical Center of Göttingen, Göttingen, Germany; ^4^Cluster of Excellence “Multiscale Bioimaging: From Molecular Machines to Networks of Excitable Cells,” University of Göttingen, Göttingen, Germany

**Keywords:** cardiomyocytes, differentiation, MLC2v, smartphone, stem cells

## Abstract

Patient-derived induced pluripotent stem cells (iPSCs) can be differentiated into atrial and ventricular cardiomyocytes to allow for personalized drug screening. A hallmark of differentiation is the manifestation of spontaneous beating in a two-dimensional (2-D) cell culture. However, an outstanding observation is the high variability in this maturation process. We valued that contractile parameters change during differentiation serving as an indicator of maturation. Consequently, we recorded noninvasively spontaneous motion activity during the differentiation of male iPSC toward iPSC cardiomyocytes (iPSC-CMs) to further analyze similar maturated iPSC-CMs. Surprisingly, our results show that identical differentiations into ventricular iPSC-CMs are variable with respect to contractile parameters resulting in two distinct subpopulations of ventricular-like cells. In contrast, differentiation into atrial iPSC-CMs resulted in only one phenotype. We propose that the noninvasive and cost-effective recording of contractile activity during maturation using a smartphone device may help to reduce the variability in results frequently reported in studies on ventricular iPSC-CMs.

**NEW & NOTEWORTHY** Differentiation of induced pluripotent stem cells (iPSCs) into iPSC-derived cardiomyocytes (iPSC-CMs) exhibits a high variability in mature parameters. Here, we monitored noninvasively contractile parameters of iPSC-CM during full-time differentiation using a smartphone device. Our results show that parallel maturations of iPSCs into ventricular iPSC-CMs, but not into atrial iPSC-CMs, resulted in two distinct subpopulations of iPSC-CMs. These findings suggest that our cost-effective method may help to compare iPSC-CMs at the same maturation level.

## INTRODUCTION

Cardiomyocytes (CMs) derived from human inducible pluripotent stem cells (iPSCs) represent a powerful model to study aspects of human cardiac muscle physiology avoiding the need for human donor hearts (1). In addition, iPSCs are available from patients with heart disease and may therefore allow the modeling of individual patient-specific therapy by retaining the genetic background and exhibiting the disease phenotype in vitro (2).

The standard process of differentiation of CMs from iPSCs involves the time-dependent application of modulators of canonical WNT signaling, such as CHIR and IWP2, followed by a time interval of metabolic selection with lactate to achieve about ∼90% purity of iPSC-CMs toward the ventricular phenotype (3). Typically, the first sign of successful differentiation to CMs is the appearance of contractile activity in two-dimensional (2-D) cultures within 2 wk (4–6). A robust maturation process is usually assumed to be achieved at *days 60–120* since the cultures strongly express cardiac-specific markers such as connexin-43 and β-myosin heavy chain (7).

However, it was frequently noticed that there is a high variability in the output of the maturation process toward iPSC-CMs (8) even with respect to contractility (9). Reasons for the variability include iPSC lines with chromosomal aberration (10), the use of different serum media (11), and the coexistence of cardiomyocytes and noncardiomyocytes at varied proportions during cardiac differentiation (12).

Approaches to generate less variably but more mature iPSC-CMs include >100-day long-term culture (7), biomechanical or electrical stimulation (13, 14), or coculture with non-CM and/or extracellular matrix components (15–17). Since those experimental approaches are *1*) complex to perform, *2*) expensive, *3*) time-consuming, and *4*) in part invasive, we aimed at a more individualized noninvasive method to determine the status of iPSC-CMs during maturation to compare iPSC-CMs from the same developmental stage for further analysis. For this purpose, we used video-based analysis of contractile activity in monolayers of iPSC-CMs that has been previously used to detect proarrhythmic and anti-arrhythmic drug effects (18, 19). Video-based analysis lacks information on the electrophysiological parameters, but is a promising approach for quick and reliable screening of the mechanical beating behavior of cardiomyocytes since contraction and relaxation result in the detectable displacement of membrane and organelle structures (20) and reflects functional characteristics of iPSC-CM monolayers (21) and engineered cardiac tissue constructs (22).

Accordingly, we monitored noninvasively contractile activity during the time course of maturation in parallel and in series. We focused on a male iPSC line since gender differences with respect to contractility were reported only after the external application of sex hormones or drugs to iPSC-CMs (23, 24). Surprisingly, our data revealed that, even within the same maturation procedure, individual samples behave differently with respect to contractile activity. We suggest that noninvasive monitoring of contractile activity using a standard smartphone device represents an economical and powerful tool to determine the individual maturation status of ventricular- and atrial-derived iPSC-CMs. This procedure may help to compare characteristics of iPSC-CMs at a similar maturation status thereby reducing the reported variability in the maturation outcome of the iPCS-CM differentiation process.

## MATERIAL AND METHODS

### Handling of Human iPSC-Derived Cardiomyocytes

Experimental protocols were approved by the ethics committee of the University Medical Center Göttingen (10/9/15). The human iPSC line isWT1.Bld2 (GOEi014-A.2, 31a old male donor) was differentiated into ventricular iPSC-CMs and atrial iPSC-CMs using a cell density of ∼1.5 × 10^6^ cells as described previously (3, 25). In brief, differentiation was initiated at 80–90% confluence in Geltrex-coated six-well plates with cardio differentiation medium composed of RPMI 1640 with Glutamax and HEPES (Thermo Fisher Scientific), 0.5 mg/mL of human recombinant albumin, and 0.2 mg/mL of l-ascorbic acid 2-phosphate, and sequential treatment with 4 μM CHIR99021 (Merck Millipore) for 48 h and then 5 μM IWP2 (Merck Millipore) for 48 h. The medium was changed to RPMI 1640 with Glutamax, HEPES, and 2% B27 (Thermo Fisher Scientific) at *day 8*. Metabolic CM selection was performed using RPMI 1640 without glucose (Thermo Fisher Scientific), 0.5 mg/mL of human recombinant albumin, 0.2 mg/mL of l-ascorbic acid 2-phosphate, and 4 mM lactate (Sigma-Aldrich) for 5 days starting between *days 19* and *22*. The differentiation toward atrial iPSC-CMs followed the protocol described using 1 µM retinoic acid for selection (25). Afterward, iPSC-CMs were cultured at least to *day 43* for further maturation and used in the experiments at the time points indicated. Supplemental Fig. S1 (all Supplemental data and legends are available at: https://doi.org/10.6084/m9.figshare.24759609.v1 and https://doi.org/10.6084/m9.figshare.24943824) shows an overview of the study design. In total, six and five six-well plates were differentiated in series to ventricular and atrial iPSC-CMs, respectively. One plate of atrial iPSC-CM was lost due to contamination. There was no replating during the recordings to avoid changes in contractility and MLC2v by this process as recently reported (26).

### Recording of Motion Activity

Motion activity was measured in each well of the six-well plates at the indicated time points, usually starting at *day 9*. For this purpose, each plate was removed from the incubator (37°C) and placed on an inverted microscope (Primovert, Zeiss, Oberkochen, Germany) equipped with a ×40 objective. Motion was recorded in the slow motion mode (120 fps) in each well for at least 10 s using a commercial smartphone fixed to the microscope ocular by a commercial smartphone digiscoping adapter as described previously (27). The recording of each six-well plate took usually ∼2 min. The position of the recorded area was manually marked for each well to ascertain repetitive recordings of the same region of interest (ROI). However, we did not observe statistically significant differences in motion activity if we recorded motion activity from different ROI in the same well (Supplemental Fig. S2) indicating homogeneous contractile activity in each well. Temperature was measured using a commercial infrared thermometer at the end of the recordings and amounted to 35 ± 1°C indicating no significant drop of temperature during motion recordings. The obtained movies were transferred from the smartphone to a standard personal computer with 16 GB memory for further analysis.

### Motion Analysis Using Myocyter Plugin

Movies recorded by the smartphone were converted by open-source software (https://pazera-free-mp4-to-avi-converter) and then analyzed using the Myocyter plugin for ImageJ (https://imagej.nih.gov/ij/) which was proposed to be superior to the MuscleMotion plugin (27, 28). In brief, regions of interest were selected manually and the Myocyter plugin was used according to the manufacturer instructions (27) to get the traces and plots of the motion activity from the selected ROI.

### Analysis of Motion Parameters Using Spiky Plugin

Traces of motion activity obtained by Myocyter were normalized using GraphPad Prism 9 (GraphPad Software Inc.) and then fed into the Spiky plugin for ImageJ as described previously (29). Normalization was used to correct for differences in motion amplitudes to get comparable motion parameters from each single recording since comparing amplitudes from different experiments was found less reliable (27). From each recording, times of peak to peak [pk2pk, corresponding to heartbeat variability (30)], the full width of half maximum (FWHM), time from baseline to peak (time2pk), and the τ value of the decay time from half maximum to baseline were obtained from analysis by the Spiky plugin and averaged from at least three motion events. Negative τ values were excluded from the analysis. The outcome of the analysis by the Spiky plugin was verified manually for a subset of traces using Origin Software (Origin 2023, www.originlab.com).

### Confocal Imaging of iPSC-CM

Human iPSC-CMs were mechanically collected from single wells and transferred to glass coverslips (Ø 18 mm, width 1.5, Menzel), fixed (Roti-Histofix 4%, Carl Roth) at room temperature (RT) for 20 min, and blocked with stem cell blocking solution at 4°C overnight. Cells were incubated with primary antibodies (anti-MLCv, Proteintech, Cat. No. 10906-1-AP, 1:200; anti-MLCa, SynapticSystems, Cat. No. 311011, 1:500; Alexa Fluor 488 Phalloidin, Thermo Fisher, Cat. No. A12379, 1:200) and diluted in stem cell blocking solution at 4°C overnight. Then, coverslips were washed thrice with stem cell blocking solution and finally incubated with secondary antibodies in stem cell blocking solution at RT for 1 h (Abberior STAR 635 P aRb, Cat. No. 53399; Abberior STAR 580 aMo, Cat. No. 52403; 1:200 each). Images were acquired with a confocal laser scanning microscope (LSM880, Carl Zeiss, Oberkochen, Germany) equipped with a Plan-Apochromat 40/water objective using 8× line averaging. Intensities of the respective markers were normalized to the minimal and maximal intensity during image acquisition. To reduce investigator bias, six images per coverslip were selected that show a prominent phalloidin signal. Afterward, these images were monitored for MLC2v and MLC2a signals. Image analysis was performed by calculating total intensities for each marker and image regardless of cellular structures using ImageJ and normalized to the intensity of the phalloidin signal.

### Analysis of Protein Expression by Immunoblotting

Immunoblotting was performed as described previously (31). Briefly, human iPSC-CMs were mechanically collected from single wells as described. Cells were pooled from two wells and homogenized in ice-cold RIPA buffer using a Potter homogenizer (RW20 digital, IKA). The homogenate was centrifuged (10,000 *g* for 10 min at 4°C) and the supernatant was used to determine the protein concentration (Pierce BCA Protein Assay Kit; Thermo Fisher Scientific). For immunoblotting, 30 μg of cleared homogenate was loaded per lane onto a 4–20% Tris-glycine gradient gel (Novex 4–20% Tris-glycine, Thermo Fisher Scientific) and resolved by SDS gel electrophoresis at constant 200 V for 45 min. Proteins were transferred onto PVDF membranes (0.45 mm, Immobilon-FL, Merck Millipore) using an electrophoretic transfer cell (Mini Trans-Blot Electrophoretic Transfer Cell, Bio-Rad) at constant 100 V for 1 h in transfer buffer at 4°C. PVDF membranes were incubated for 1 h in 5% wt/vol nonfat milk (Milkpowder, Roth) in Tris-buffered saline with 0.05% vol/vol Tween (Tween 20, Sigma-Aldrich). Then, PVDF membranes were decorated with the primary antibodies (anti-Serca2a, Badrilla Cat. No. A010-20, 1:1,000; anti-MLCv, Proteintech, Cat. No. 10906-1-AP, 1:1,000; anti-MLCa, SynapticSystems Cat. No. 311011, 1:1,000; anti-GAPDH, Biotrend, Cat. No. 5G4 Mab 6C5, 1:8,000) at 4°C overnight. Thereafter, membranes were washed thrice with PBS (pH 7.4, without Ca^2+^ and Mg^2+^, Gibco) and incubated with fluorescent anti-mouse or anti-rabbit secondary antibodies at a dilution of 1:15,000 for at least 1 h at RT (IRDye LI-COR). Fluorescence signals were monitored with an Odyssey CLx imaging system (LI-COR) and band intensities were analyzed with Image Studio Lite Version 5.2 (LI-COR). Intensities were normalized to GAPDH intensities for the respective lane. For correlation analysis, intensities of anti-MLC2v signals were plotted against the respective mean of motion frequencies obtained from the respective two wells.

### Statistical Analysis

Statistical and correlation analysis was performed using Prism 9 software (GraphPad Software Inc). Data are presented as means ± SE. Numbers indicate the number of analyzed experiments. Statistically significant differences were determined either by paired or unpaired Student’s *t* test (2 groups) or by one-way ANOVA followed by a Turkey’s post hoc test (multiple groups). All data sets passed a test for normality. *P* < 0.05 was considered statistically significant.

## RESULTS

### Spontaneous Motion Activity in Ventricular iPSC-CMs

**Figure 1.**
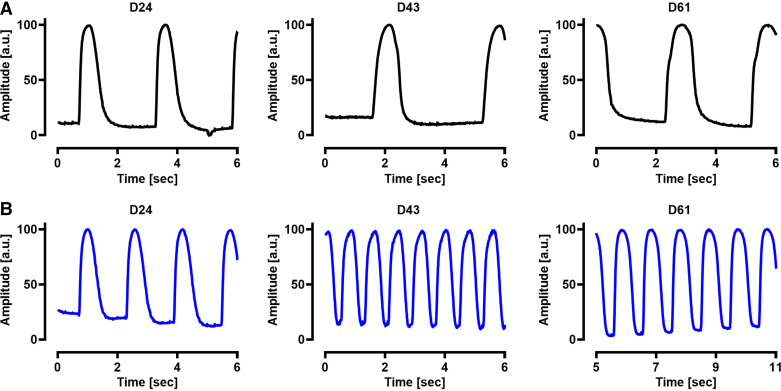
Motion activity during differentiation of induced pluripotent stem cells (iPSC) into ventricular iPSC cardiomyocytes (CMs). *A* and *B*: spontaneous motion activity in two wells of ventricular iPSC-CMs from the identical differentiation procedure obtained at *days 24*, *43*, and *61* after the start of the differentiation. *A*: cells (black) show two to three motion events within 6 s at the days shown. *B*: cells (blue) show four motion events at *day 24* and six to seven motion events at *days 43* and *61*. Motion activity was assigned to a “slow beating” behavior (*A*) and a “fast beating” behavior (*B*) of ventricular iPSC-CMs.

iPSCs were differentiated in six-well plates to the ventricular subtype as previously described (3, 25). Spontaneous motion activity was monitored in each well every 2–3 days starting at *day 9*. Figure 1, *A* and *B*, shows the motion activity in two wells of the identical plate recorded on *days 24*, *43*, and *61* after the start of the differentiation procedure. In Fig. 1*A*, the motion activity shows a slow frequency and a long duration of the motion events whereas in Fig. 1*B*, the motion activity displays a high frequency and a short duration of the motion events. Consequently, we assigned the motion pattern of Fig. 1*A* to a “slow beating” behavior (Supplemental Movie S1) and the pattern of Fig. 1*B* to a “fast beating” behavior of the respective well-containing ventricular iPSC-CMs (Supplemental Movie S2).

**Figure 2.**
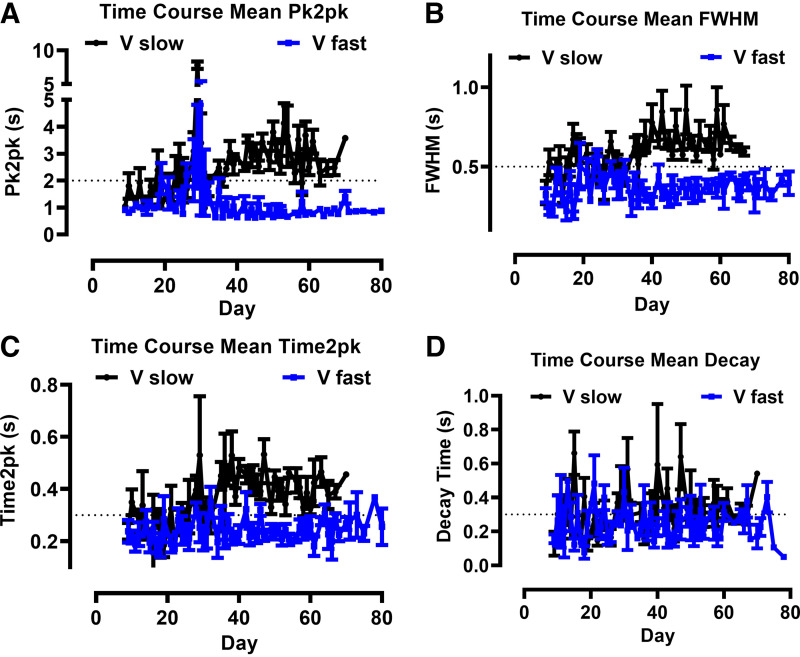
Averaged time courses of motion parameters during the differentiation of induced pluripotent stem cells (iPSC) into ventricular iPSC cardiomyocytes (CMs). The obtained time courses of peak to peak time (pk2pk; *A*), full width at half maximum (FWHM; *B*), time to peak (time2pk; *C*), and decay time (*D*) are depicted. Motion parameters that were assigned to a “slow beating” behavior (black) or to a “fast beating” behavior (blue) were averaged separately. Lactate was omitted in the medium for 5 days starting between *days 18* to *22* according to the differentiation protocol. Dotted lines represent the manually assigned thresholds with 2 s for *A*, 0.5 s for *B*, and 0.3 s for *C* and *D*. Data are shown as means ± SE with *n* = 3–10 wells (black) and *n* = 3–7 wells (blue) and correspond to motion activity from 36 single wells out of six different differentiations in 6-well plates (i.e., 6 different batches of ventricular iPSC-CMs).

**Figure 3.**
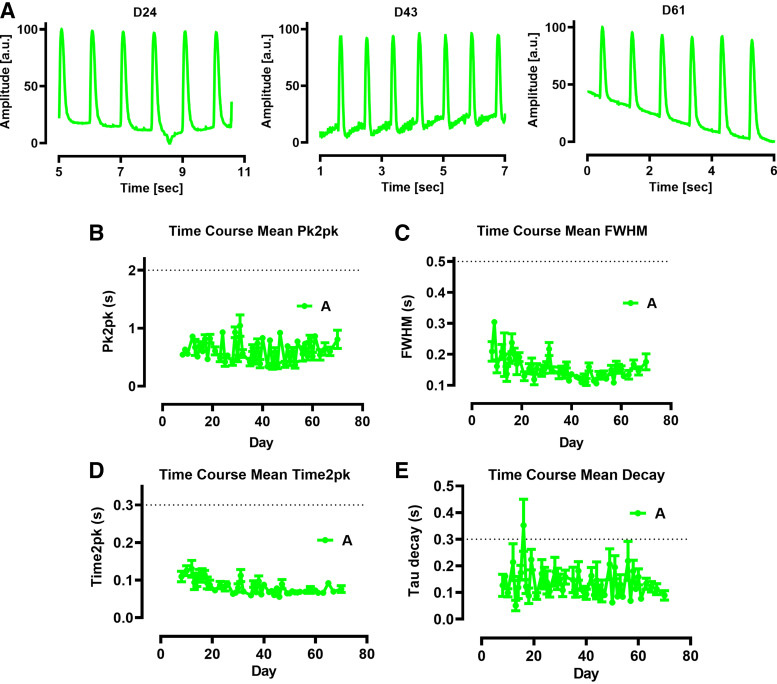
Motion activity during differentiation of induced pluripotent stem cells (iPSC) into atrial iPSC cardiomyocytes (CMs). *A*: spontaneous motion activity in 1 well of atrial iPSC-CMs from the identical differentiation procedure obtained at *days 24*, *43*, and *61* after the start of the differentiation. *B–E*: averaged time courses of motion parameters during the differentiation of iPSC to atrial iPSC-CMs. The obtained time courses of peak-to-peak times (pk2pk; *B*), full width at half maximum (FWHM; *C*), time-to-peak (time2pk; *D*), and decay time (*E*) are depicted. Lactate was omitted in the medium for 5 days starting between *days 18* to *22* according to the differentiation protocol. Dotted lines represent the assigned thresholds for ventricular iPSC-CMs with 2 s for *B*, 0.5 s for *C*, and 0.3 s for *D* and *E*. Data are shown as means ± SE with *n* = 6–16 (green) and correspond to motion activity from 30 single wells out of five different differentiations in 6-well plates (i.e., 5 different batches of atrial iPSC-CMs). No interwell differences in the parameters were observed indicating a similar atrial phenotype in all wells.

**Figure 4.**
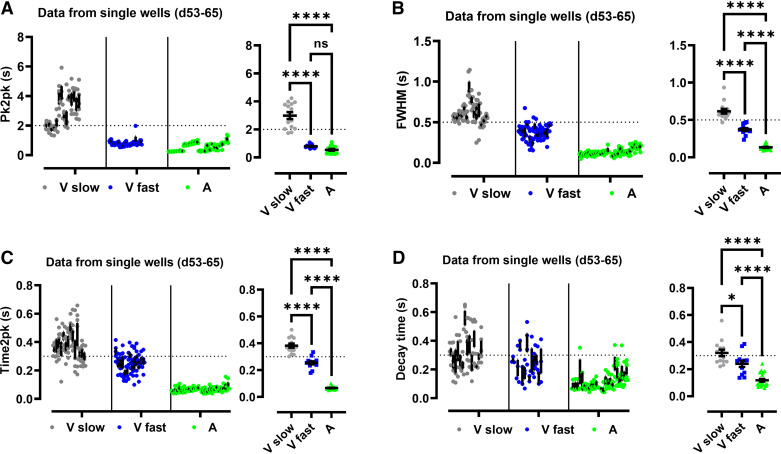
Comparison of motion parameters during differentiation of induced pluripotent stem cells (iPSC) to iPSC cardiomyocytes (CMs). Values are means ± SE of peak to peak time (pk2pk; *A*), full width at half maximum (FWHM; *B*), time to peak (time2pk; *C*), and decay time (*D*) are superimposed that were obtained in single wells during the time interval from *day 53* to *day 65*. Dots correspond to wells from “slow beating” ventricular iPSC-CMs (gray), from “fast beating” ventricular iPSC-CMs (blue), and from atrial iPSC-CMs (green). Data correspond to motion activity from 30 and 36 single wells out of 5 and 6 different differentiations (batches) in 6-well plates for atrial and ventricular iPSC-CMs, respectively. Ordinary one-way ANOVA followed by Tukey’s multiple comparisons test was applied to the mean values for statistical analysis of the data sets, respectively [with black/blue ****P = 5 × 10^−7^, ns, green/blue *P* = 0.345 and ****, green/black *P* = 5 × 10^−10^ (*A*); black/blue ****, *P* = 2 × 10^−6^, ****, green/blue *P* = 2 × 10^−7^ and ****, green/black *P* = 2.3 × 10^−13^ (*B*); black/blue ****, *P* = 3 × 10^−6^, green/blue *P* = 3.1 × 10^−9^ and ****, green/black *P* = 5 × 10^−14^ (*C*); and black/blue *, *P* = 0.016, ****, green/blue *P* = 0.0003 and ****, green/black *P* = 5.2 × 10^−8^ (*D*)].

Figure 2, *A–D*, summarizes the time courses of motion activities of all experiments with respect to the parameters *1*) frequency (depicted as time between two peaks, pk2pk, Fig. 2*A*), *2*) duration of contraction (depicted as full width at half-maximal amplitude, FWHM, Fig. 2*B*), *3*) development of contraction (depicted as time to peak, time2pk, Fig. 2*C*), and *4*) time of relaxation (shown as decay from half maximum to baseline, decay time, Fig. 2*D*). Motion activities of wells assigned to the “slow” and “fast beating” phenotype were averaged separately. The individual time courses of the parameters for four independent differentiation procedures are shown in Supplemental Fig. S3. An increase in pk2pk and time2pk was prominent during the 5-day-lasting substitution of glucose by lactate starting between *days 18* and *22* in all experiments. All values were larger in the “slow” compared with the “fast beating” group of ventricular iPSC-CMs. The ratios of the phenotypes (“slow” vs. “fast”) during the identical differentiations in the six-well format were found to be 3:3 to 5:1. Pk2pk values did not differ during the recordings indicating a homogeneous rhythmic activity in each well examined. Arrhythmic events evidenced by double beats were randomly observed in ∼2% of the recordings and excluded from analysis. Strikingly, once the “slow” versus “fast” phenotype of the differentiated wells was established early in the differentiation phase, it remained stable during continuous culture and maturation, i.e., we did not observe a switch between the phenotypes during the time course of our experiments. In summary, we found two patterns of contractile activity reflected by spontaneous motion activity in ventricular iPSC-CMs during identical differentiation procedures suggesting the presence of distinct subpopulations of ventricular iPSC-CMs that establish early in maturation. In invasive experiments, the contractile activity was accelerated by β-adrenergic stimulation using isoproterenol (1 µM) and reduced by muscarinergic stimulation using carbachol (10 µM) demonstrating the presence of the respective vegetative signaling pathways in the differentiated cultures (Supplemental Fig. S4).

### Spontaneous Motion Activity in Atrial iPSC-CMs

iPSCs were differentiated in six-well plates to the atrial subtype as previously described (3). Figure 3*A* shows the motion activity in one well of one plate recorded on *days 24*, *43*, and *61* after the start of the cell culture (Supplemental Movie S3). We did not observe a clear-cut difference in the motion activity in the well shown during the time course of the experiment.

As shown above for ventricular iPSC-CMs, Fig. 3, *B–E*, summarizes the time courses of motion activities of all experiments on atrial iPSC-CMs with respect to the parameters pk2pk (Fig. 3*B*), FWHM (Fig. 3*C*), time2pk (Fig. 3*D*), and decay time (Fig. 3*E*). All parameters showed a minor variability until *day 40* and displayed afterward values that were more constant. The individual time courses of the parameters for four independent differentiation procedures are shown in Supplemental Fig. S5. In summary, we detected only one phenotype of spontaneous contractile activity in atrial iPSC-CMs during the maturation procedure.

### Comparison of Motion Activity in Ventricular and Atrial iPSC-CMs

Figure 3 superimposes all individual parameters of motion activity, i.e., of pk2pk (Fig. 4*A*), FWHW (Fig. 4*B*), time2Pk (Fig. 4*C*), and decay times (Fig. 4*D*) that were collected in the time interval from *day 53* to *day 65*. All values were significantly larger in the “slow” compared with the “fast beating” group of ventricular iPSC-CMs. Values of pk2pk were not different between atrial iPSC-CMs and the “fast beating” group of ventricular iPSC-CMs but clearly lower compared with the “slow beating” group of ventricular iPSC-CMs (Fig. 4*A*). Values of FWHM, time2pk, and decay time in atrial iPSC-CM were significantly lower compared with the respective values from ventricular iPSC-CMs both corresponding to the “fast” and “slow beating” group (Fig. 4, *B–D*). Obviously, the overall motion activity of the “fast beating” group of ventricular iPSC-CMs was in between the motion activity of the “slow beating” group of ventricular iPSC-CMs and the atrial iPSC-CMs indicating a distinct subpopulation of ventricular iPSC-CM with unique functional characteristics. No statistical differences were found for the parameters of atrial iPSC-CMs between the time intervals *days 35–44* and *days 56–65* (data not shown).

### Immunofluorescence of MLC2v and MLC2a in Ventricular and Atrial iPSC-CMs

**Figure 5.**
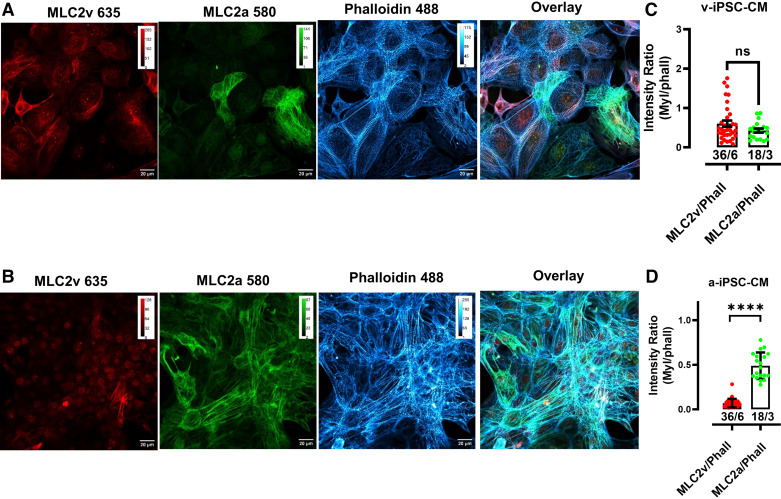
Confocal imaging of induced pluripotent stem cells cardiomyocytes (iPSC-CMs) immunostained for MLC2v, MLC2a, and phalloidin. *A* and *B*: representative image of ventricular (*A*) and atrial (*B*) iPSC-CMs decorated with anti-MLC2v (red), anti-MLC2a (green), and phalloidin (magenta). The overlay of all three signals is depicted on the right. MLC2v-positive as well as MLC2a-positive iPSC-CMs were found in ventricular and atrial iPSC-CMs. *C*: comparison of total MLC2v/phalloidin ratios with total MLC2a/phalloidin ratios in ventricular iPSC-CM. Data points correspond to values from 6 images/well with six different wells for MLC2v:phalloidin and three different wells for MLC2a/phalloidin out of 1 batch of iPSC-CM. Statistical analysis was performed by unpaired Student’s *t* test with ns, *P* = 0.13. *D*: comparison of total MLC2v:phalloidin ratios with total MLC2a:phalloidin ratios in atrial iPSC-CM. Data points correspond to values from 6 images/well with six different wells for MLC2v/phalloidin and three different wells for MLC2a/phalloidin out of one batch of iPSC-CM. Statistical analysis was performed by unpaired Student’s *t* test with ****P < 1 × 10^−15^.

iPSC-CMs from six wells of one differentiation were transferred to glass coverslips at the end of motion analysis for use in immunofluorescence. iPSC-CMs were stained with phalloidin/f-actin as a sarcomeric marker and with both, an atrial and a ventricular cardiac muscle marker, i.e., MLC2a and MLC2v. Notably, MLC2a is also found to be expressed in immature ventricular iPSC-CMs. Figure 5 shows a typical staining of ventricular iPSC-CMs (Fig. 5*A*) and atrial iPSC-CMs (Fig. 5*B*) with MLC2v, MLC2a, phalloidin, and the overlay of all three markers. We found both MLC2v and MLC2a positive cells in the ventricular iPSC-CMs and predominantly MLC2a positive cells in the atrial iPSC-CMs. For overall quantification, we analyzed the total marker intensities for each image and related them to the total intensity of phalloidin. Intensity ratios of MLC2v/phalloidin and MLC2a/phalloidin were statistically not different in ventricular iPSC-CMs (Fig. 5*C*) indicating a similar distribution of immature and more mature ventricular iPSC-CMs within the cultures. In contrast, intensity ratios were significantly larger for MLC2a/phalloidin versus MLC2v/phalloidin in atrial iPSC-CMs (Fig. 5*D*) indicating an increased distribution of the atrial marker with respect to the sarcomeric marker phalloidin.

The sarcomeric pattern in ventricular iPSC-CM confirmed the presence of immature (MLC2a-positive) as well as of more mature (predominantly MLC2v-positive) ventricular iPSC-CMs in the cultures (Supplemental Fig. S6). These findings indicate that both ventricular-like and atrial-like CMs are present in the ventricular iPSC-CMs as shown previously (32). In atrial iPSC-CMs, mature (MLC2a positive) as well as poor mature (MLC2v and MLC2a positive) cells were detected indicating overall a more homogenous pattern of atrial iPSC-CMs. Interestingly, the phalloidin-signal followed a *Z*-line-like structure in both atrial and ventricular iPSC-CMs that was validated by an α-actinin costaining (Supplemental Fig. S7).

### Correlation of MLC2v/Phalloidin Intensity with Motion Frequency in iPSC-CMs

**Figure 6.**
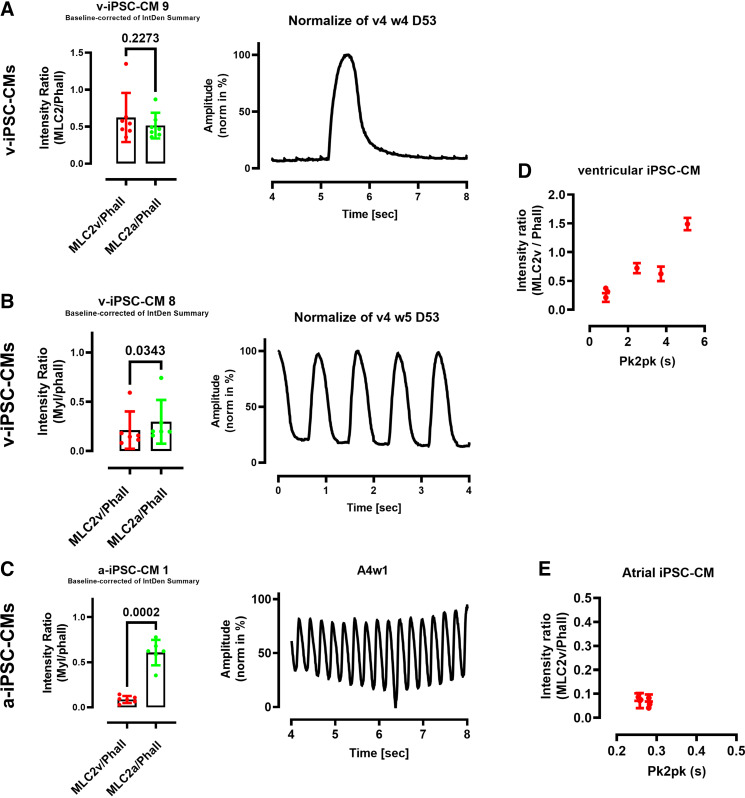
Comparison of total MLC2v:phalloidin ratios with motion activity in the respective wells from induced pluripotent stem cells cardiomyocytes (iPSC-CMs). *A–C*: comparison of total MLC2v:phalloidin ratios with total MLC2a:phalloidin ratios obtained from two different wells of ventricular iPSC-CMs (*A* and *B*) and from one well of atrial iPSC-CMs (*C*) with the corresponding motion activity recorded before performing the protocol for immunofluorescence. Data points correspond to values from 6 images/well out of one batch of iPSC-CMs. Statistical analysis was performed by unpaired Student’s *t* test with the indicated *P* values. *D*: correlation analysis of total mean MLC2v:phalloidin ratios obtained from six wells out of one batch of ventricular iPSC-CMs with the respective peak to peak (pk2pk) values obtained by motion analysis before performing the protocol for immunofluorescence with Pearson *r* = 0.9187 and *P* = 0.0096. *E*: correlation analysis of total mean MLC2v:phalloidin ratios obtained from six wells of atrial iPSC-CM out of one batch with the respective pk2pk values obtained by motion analysis before performing the protocol for immunofluorescence with Pearson *r* = −0.5888 and ns, *P* = 0.2188.

Ratios of the MLC2v/phalloidin signals were correlated to the motion activity recorded before starting the protocol for immunofluorescence. Figure 6, *A* and *B*, shows the MLC2v/phalloidin and MLC2a/phalloidin ratios in two different wells from ventricular iPSC-CMs together with the corresponding motion activities. From these figures, we hypothesized that the MLC2v/phalloidin ratio may inversely correlate with the motion frequency. Therefore, we plotted the respective beat frequency values (depicted as pk2pk) to the averaged MLC2v/phalloidin ratios obtained from six wells (Fig. 6*D*). A correlation analysis resulted in a significant correlation between the MLC2v/phalloidin ratio and the pk2k values (Pearson *r* = 0.9187; *P* = 0.0096). This finding indicates that the “slow beating” group of ventricular iPSC-CMs exhibit more mature ventricular-like sarcomeric structures than the “fast” beating group. Similar results were obtained for the correlation between the MLC2v/phalloidin ratio and the respective FWHM and time2pk values but not for the decay time values (Supplemental Fig. S8). Figure 6*C* shows the MLC2v/phalloidin and MLC2a/phalloidin ratios in one well from atrial iPSC-CM together with the corresponding motion activity. In atrial iPSC-CMs, single wells showed about fivefold higher MLC2a/phalloidin ratio compared with the MLC2v/phalloidin ratio. A correlation analysis resulted in no correlation between the MLC2v/phalloidin ratio and the pk2k values (Pearson *r* = −0.588; n.s., *P* = 0.2188; Fig. 6*E*) confirming the more consistent phenotype of atrial versus ventricular iPSC-CMs.

### Western Blot Analysis of Ventricular and Atrial iPSC-CMs

**Figure 7.**
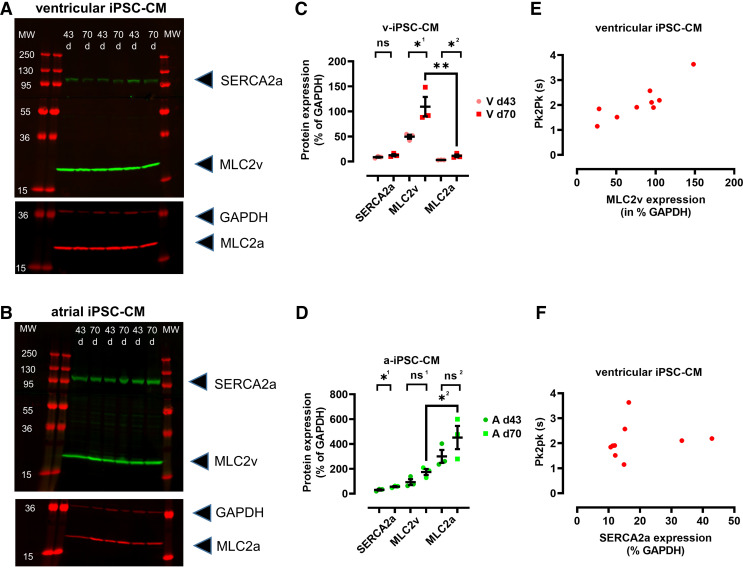
Immunoblot of induced pluripotent stem cells cardiomyocytes (iPSC-CMs) differentiated to *day 43* or *day 70*. *A* and *B*: immunoblot of ventricular (*A*) and atrial (*B*) iPSC-CMs. Signals for SERCA2a, MLC2v, MLC2a, and GAPDH are shown after normalization to the respective maximal intensities. Cells were collected after maturation to *day 43* and to *day 70* from two different 6-well plates (i.e., from two batches), respectively. Cells from two wells were pooled and lysates were placed on the gel in an alternating procedure for ventricular (*A*) and atrial (*B*) cells as indicated. Blots were imaged in sequence after the application of the respective secondary antibodies. For clarity, only the lower part of the second image is shown for ventricular and atrial iPSC-CMs, respectively. *C* and *D*: quantification of the immunoblots shown in *A* and *B*. Intensities were normalized to the GAPDH signal. Statistical analysis was performed by unpaired Student’s *t* test resulting for *C* with ns, *P* = 0.15; *^1^*P* = 0.04, *^2^*P* = 0.03, **P = 0.0075 and for *D* with *^1^*P* = 0.02; ns^1^ *P* = 0.07; ns^2^ *P* = 0.23; *^2^*P* = 0.04. *E* and *F*: correlation analysis of the MLC2v:GAPDH signals (*E*, pooled from 2 wells) and the SERCA2a:GAPDH signals (*F*, pooled from 2 wells) with the peak to peak (pk2pk) values obtained by motion analysis (averaged from the 2 respective wells) in ventricular iPSC-CM with Pearson *r* = 0.86 and *P* = 0.002 (*E*) and Pearson *r* = 0.15 and ns, *P* = 0.7 (*F*).

Differences in protein expression during maturation, especially for MLC2v and MLC2a, have been reported for ventricular and atrial iPSC-CM (3). Therefore, we comparatively performed well-specific Western blots for ventricular and atrial iPSC-CMs maturated to *day 43* and *day 70*. Figure 7 shows Western blots for ventricular (Fig. 7*A*) and atrial iPSC-CM (Fig. 7*B*) decorated with antibodies against SERCA2a as a cardiac marker, MLC2v, MLC2a, and, finally, GAPDH as a loading control. All four proteins were detected in our samples from both ventricular and atrial iPSC-CMs. The quantification of the Western blot for ventricular iPSC-CM is shown in Fig. 7*C*. In our samples from ventricular iPSC-CMs, MLC2v expression was significantly enlarged compared with MLC2a expression (∼3-fold) confirming the major presence of the ventricular phenotype. In addition, MLC2v expression was significantly larger in samples from *day 70* maturation compared with *day 43* maturation indicating that maturation of ventricular iPSC-CMs is incomplete at *day 43* as suggested (33, 34). Expression of SERCA2a was not different between *days 43* and *70* in ventricular iPSC-CM. For atrial iPSC-CM, quantification of the Western blot revealed that MLC2a expression is significantly enlarged compared with MLC2v expression confirming the major presence of the atrial phenotype in our cultures (Fig. 7*D*). MLC2a expression was not different between *day 43* and *day 70* of maturation but SERCA2a expression was significantly larger at *day 70* compared with *day 43* in atrial iPSC-CM. These findings confirm that the expression of the markers MLC2v and MLC2a correspond to the ventricular and atrial phenotype at greater than *day 60*, respectively. Interestingly, the maturation of atrial iPSC-CMs at *day 43* was found to be more complete than that of ventricular iPSC-CMs.

### Correlation of MLC2v Expression with Motion Frequency in Ventricular iPSC-CMs

Expression levels of MLC2v and SERCA2a (normalized to GAPDH expression) were determined for single wells and correlated to the last motion activity recorded in these wells before starting the protocol for immunoblotting. The obtained correlations for MLC2v are shown in Fig. 7*E* and those for SERCA2a in Fig. 7*F*. Analysis of the correlations resulted in a significant correlation for MLC2v and beat frequency (shown as pk2pk) values (*P* = 0.002) but no correlation between SERCA2a and pk2pk (*P* = 0.7). This finding implicates that the “fast beating” group of ventricular iPSC-CMs exhibited lower expression levels of MLC2v than the “slow beating” group of ventricular iPSC-CMs, similar to the findings described above for MLC2v/phalloidin intensities. Moreover, our data indicate the early manifestation of distinct subpopulations of more mature and immature cultures in a well to well-dependent manner.

## DISCUSSION

The present study confirms that noninvasive motion analysis using a standard smartphone device serves as a cost-effective and reliable tool to assess the maturation status of iPSC-CMs (19, 20, 27). Our results suggest that individual long-term differentiation procedures resulted in different maturation phenotypes, adding a previously unnoticed complexity in iPSC-CM maturation, especially for ventricular iPSC-CMs with respect to contractility and MLC2v expression that may contribute to the reported variability in iPSC-CM differentiation.

Motion activity of iPSC-CMs monitors cardiac contraction and relaxation by light-detectable displacements of cell structures based on pixel intensity movement (20, 27). Indeed, motion analysis on human embryonic stem cell-derived CM was used to quantify the total amount of change in cell morphology over time to generate a similarity matrix that detects subtle changes in the contractile kinetics in response to isoproterenol and verapamil (35). Furthermore, the latter study shows that motion events clearly correlate with force generation and Ca^2+^ signals. In addition, in iPSC-CMs, calcium signals and motion were shown to go hand in hand as is expected (36). Likewise, motion recordings have been used on conventional monolayers of iPSC-CMs for simultaneously assessing the mechanical and electrical actions of drugs like amiodarone (18, 37). Although the latter approaches combine motion recordings with invasive techniques, i.e., multielectrode array recordings or loading with intracellular Ca^2+^ indicators, motion recording as a stand-alone approach represents a quick and reliable method for simplified drug screening efforts (20, 35).

Spontaneous contractile activity is a hallmark in the cardiomyogenesis of iPSC toward iPSC-CMs occurring within 2 wk after the start of the differentiation (5). Subsequently, differences in the frequency have been taken as a strong indicator for the successful differentiation of iPSCs toward ventricular-like and atrial-like iPSC-CMs (3). During the maturation process, it has frequently been noted that spontaneous activity, i.e., frequency, was diminished in the late stage (*days 60* to *120*) compared with early stage (*days 20–40*) ventricular iPSC-CMs (7, 38) whereas no difference was observed between early stage (*day 15*) and middle stage (*day 45*) ventricular iPSC-CMs (39). The reduction in frequency was accompanied by longer durations and slower contractile performance observed in late-stage iPSC-CMs (7, 40). Accordingly, our continuous monitoring of contractile activity confirmed the reduction in activity rate and longer durations of activity starting around the middle stage (*day 45*) indicating maturation to a ventricular-like phenotype of CM. Surprisingly, we noticed that only ∼50% of identical maturation procedures followed the aforementioned process whereas the remaining cultures showed only minor changes in activity rate or activity durations. Consequently, we divided these phenotypes into a “slow beating” and “fast beating” phenotype of ventricular iPSC-CMs. Furthermore, we noticed that the activity of the “fast beating” phenotype was in between the “slow beating” phenotype and the atrial iPSC-CMs. Obviously, the identical process of maturation for ventricular iPSC-CMs resulted in both, a ventricular-like and poor ventricular-like phenotype that may account for the observed high variability in the output of the maturation process (8).

The sarcomeric proteins MLC2v and MLC2a show a differential expression profile during heart development: MLC2a decreases in overall expression and is enriched in the atria whereas MLC2v expression becomes enriched within the ventricles (41, 42). The maturation of iPSC toward iPSC-CMs reflects this process (34). Immunostainings as well as immunoblottings confirmed that late-stage (*days 60*–*90*) iPSC-CMs exhibited increased MLC2v expression and decreased or unchanged MLC2a expression compared with early/middle (*day 20*–*40*) stage iPSC-CMs (33, 43–45). Subsequently, the expression of MLC2v and MLC2a served as markers for the characterization of the maturation of iPSC-CMs toward the atrial and ventricular phenotype (3). Here, we confirmed these findings showing a more pronounced expression of MLC2v in late-stage (*day 70*) compared with middle-stage (*day 43*) ventricular iPSC-CMs. Likewise, we observed a more pronounced expression of MLC2a compared with MLC2v in late-stage (*day 70*) atrial iPSC-CMs. Unexpectedly, we found a strong correlation between MLC2v expression and contractile activity on the base of single maturations. Thus, the “slow beating” group of ventricular iPSC-CMs exhibited more MLC2v expression than the “fast” beating group indicating that the strong correlation of motion activity and MLC2c expression characterizes a more maturated phenotype of ventricular iPSC-CMs within identical maturation procedures.

Late-stage (>*day 60*) iPSC-CMs show sarcomeric organization verified by immunolabeling of multiple myofilament proteins. For example, striated MLC2a labeling was observed in iPSC-CMs as well as an overlap of α-actinin and MLC2a labeling that demonstrated an alternating pattern in the sarcomeres in agreement with the known localization of MLC2a to the A-band of the sarcomere, which lies between the *Z*-lines highlighted by the α-actinin labeling (46). Likewise, coimmunolabeling of iPSC-CMs with MLC2v and α-actinin revealed an alternating repeating pattern with MLC-2v staining localized in each half of the sarcomeric A-band suggesting a degree of functional maturity of the cardiac sarcomere (32). Similar patterns were observed in both atrial and ventricular subtypes of iPSC-CMs visualized by α-actinin and F-actin staining (3). Similarly, in the present study, staining of both ventricular and atrial iPSC-CMs revealed a striated pattern with MLC2v and MLC2a, respectively, both probably localized in the sarcomeric A-band. Interestingly, in our hands, we experienced that the staining of iPSC-CMs with phalloidin, commonly used for staining actin filaments, correlates strongly with the α-actinin staining indicating the association of F-actin to the *Z*-lines in iPSC-CMs.

Since we analyzed only motion activity in single wells that consist of a 2-D sheet of iPSC-CM in a cardiac syncytium setting, analysis of isolated single iPSC-CM may give different results (47). However, our aim was to prejudge the maturation status of cultured iPSC-CMs before using them in single cells or in invasive experiments to compare cell lines from wild type (WT) or mutated iPSC-CMs at an equivalent maturation status. Unfortunately, for the moment, we do not know what factors determine the fate of ventricular iPSC-CMs after the metabolic selection to mature toward the “slow” or “fast” beating phenotype. Possible factors include an undesired variability in cell numbers of cardiomyocytes and noncardiomyocytes or different velocities in the establishment of cell-cell contacts to build up the cardiac syncytium both of which will be addressed in further studies.

Another limitation of our study is that we used only one iPSC line from a male donor. However, although differential gene expression was recently reported in male and female iPSC-CMs (48), the identified genes were not related to genes involved in cardiac contractility. Furthermore, sex differences related to X-chromosome dosage were described only to influence the differentiation ratio of iPSC toward cardiomyocytes and epicardium-derived cells (49) whereas, in the present study, we focused on the fate of differentiated iPSC-CMs only. We recognize the current sex inequality in stem cell research as stated (48) but feel that gender differences will not influence qualitatively the interpretation of our results.

In summary, standard long-term differentiation of iPSC toward ventricular iPSC-CMs resulted in two different contractile phenotypes in contrast to the differentiation of iPSC toward atrial iPSC-CMs. We suggest that noninvasive motion analysis may help *1*) to identify more reliable the iPSC-CMs phenotype of interest, especially in further studies using ventricular iPSC-CMs; *2*) to compare WT and mutated ventricular iPSC-CM lines within an equivalent maturation status; and *3*) to prove new previously published protocols, e.g., from the study by Funakoshi et al. (50), for long-term differentiation of iPSC toward pure ventricular iPSC-CMs.

## DATA AVAILABILITY

The source data are available to verified researchers upon request by contacting the corresponding author.

## SUPPLEMENTAL DATA

10.6084/m9.figshare.24759609.v1 Supplemental Figs. S1–S8: https://doi.org/10.6084/m9.figshare.24759609.v1.

10.6084/m9.figshare.24759609.v1 Supplemental Movies S1–S3: https://doi.org/10.6084/m9.figshare.24759609.v1.

10.6084/m9.figshare.24943824 Legends for Supplemental Material: https://doi.org/10.6084/m9.figshare.24943824.

## GRANTS

This work was funded by Deutsches Zentrum für Herz-Kreislaufforschung (German Centre for Cardiovascular Research) Grant 81Z0300117 and Deutsche Forschungsgemeinschaft (DFG, German Research Foundation; under Germany’s Excellence Strategy) Grant EXC 2067/1-390729940 (to S.E.L. and N.V.).

## DISCLOSURES

No conflicts of interest, financial or otherwise, are declared by the authors.

## AUTHOR CONTRIBUTIONS

S.E.L. and J.W.W. conceived and designed research; M.R., L.C., and J.W.W. performed experiments; M.R. and J.W.W. analyzed data; L.C., N.V., S.E.L., and J.W.W. interpreted results of experiments; J.W.W. prepared figures; J.W.W. drafted manuscript; N.V. and J.W.W. edited and revised manuscript; M.R., L.C., G.H., S.E.L., and J.W.W. approved final version of manuscript.
